# Randomized and non-randomized designs for causal inference with longitudinal data in rare disorders

**DOI:** 10.1186/s13023-021-02124-5

**Published:** 2021-11-23

**Authors:** Rima Izem, Robert McCarter

**Affiliations:** Division of Biostatistics and Study Methodology, Children’s Research Institute at Children’s National Medical Center, The George Washington University, Washington, DC USA

## Abstract

In the United States, approximately 7000 rare diseases affect 30 million patients, and only 10% of these diseases have existing therapies. Sound study design and causal inference methods are essential to demonstrate the therapeutic efficacy, safety, and effectiveness of new therapies. In the rare diseases setting, several factors challenge the use of typical parallel control designs: the small patient population size, genotypic and phenotypic diversity, and the complexity and incomplete understanding of the disorder’s progression. Repeated measures, when spaced appropriately relative to disease progression and exploited in design and analysis, can increase study power and reduce variability in treatment effect estimation. This paper reviews these longitudinal designs and draws the parallel between some new and existing randomized studies in rare diseases and their less well-known controlled observational study designs. We show that self-controlled randomized crossover and N-of-1 designs have similar considerations as the observational case series and case-crossover designs. Also, randomized sequential designs have similar considerations to longitudinal cohort studies using sequential matching or weighting to control confounding. We discuss design and analysis considerations for valid causal inference and illustrate them with examples of analyses in multiple rare disorders, including urea cycle disorder and cystic fibrosis.

## Introduction

Millions are affected by rare disorders and have an urgent need for therapy to save or improve their quality of life. Over 7000 rare diseases, disorders, illnesses, or conditions impact 25 to 30 million people in the United States, most of them are children [1]. Many rare diseases cause death in infancy or early childhood, and about 90% have no approved treatment [2]. Despite the great need for effective therapies, evaluating evidence of efficacy and safety of novel therapies in the rare disease setting is challenging in the typical paradigm of parallel-group randomized studies [3, 4]. In recognizing these challenges and the need for novel designs the US Food and Drug administration published several guidance documents in 2019 [5–7].

This paper gives an overview of study designs that rely on or exploit repeated measures for causal inference. These randomized or observational designs, when appropriately conducted and used, can alleviate some challenges in estimating treatment effects in rare disorders. Moreover, this review draws a parallel between randomized longitudinal designs and their less well known controlled observational studies counterpart. While randomized studies sit atop of the hierarchy of evidence, rigorously designed, well-controlled, and analyzed observational studies using causal inference methods can be adequate to assess the safety and effectiveness [8] of therapies in rare disorders [8]. In this paper, *the treatment effect* is the measure of efficacy or safety in a randomized clinical trial and the measure of effectiveness or safety in a comparative observational study.

When possible, we will illustrate different considerations with existing clinical trials or observational studies in rare disorders, most often with urea cycle disorders (UCD) and cystic fibrosis (CF) disorders, as they represent a broad spectrum of rare disorders from ultra-rare to rare.

## The case for longitudinal studies in rare disorders

We focus in this section on two challenges particularly salient in clinical study design in rare disorders: small population size and heterogeneity of clinical outcomes. Then, we discuss how planning and using repeated assessments of these outcomes has the potential to alleviate these challenges.

### Challenges in clinical development in rare disorders related to sample size and heterogeneity of outcomes

Designing studies with sufficient statistical power to evaluate treatment effect is challenging in rare diseases because of limited population size and high variability of outcomes. Clinical study sample sizes in rare diseases typically range from a handful of subjects to less than a few hundreds, based on disease prevalence [9]. The prevalence threshold defining ‘rare’ varies by country or organization and is in the range of 40 to 50 cases per 100,000 people [10]. This range includes incidences of CF on the higher end, with one case for every 3000–4000 births among Caucasians [11], and of ultra-rare UCD mutations on the lower end, with one case in a million births [12].

Several factors contribute to between-subject variability in outcome measurements in clinical studies. First, at any given time potential participants in clinical studies represent a cross-section of the population varying in ages and stage of disease progression. Even if age is controlled in the study, the timing of diagnosis often varies with implications for variability of medical history. For example, whereas CF is likely to be diagnosed at birth, as part of newborn screening [13], the timing of diagnosis of UCD ranges from the first few days after birth to adulthood. The timing varies due to multiple factors, including family medical history (e.g., diagnosis of family members), and varying age of presentation of symptoms such as hospitalization for hyperammonemia. Treatment of UCD disorders starts after diagnosis, and delay in therapy has downstream effects on the brain and general health.

Also, variability in genetic, geographic, and environmental factors contributes to heterogeneity in clinical outcomes. In most rare disorders, a mutation disrupts a biochemical pathway, leading to various signs and symptoms downstream. The extent and clinical impact of these disruptions vary by mutation and environmental factors. For example, some genetic mutations in the UCD reduce while others eliminate enzyme activities related to nitrogen waste disposal in the urea cycle [14–16]. For the same mutation, disease progression varies by several geographic and calendar time factors including age at diagnosis, medical practice, diet, and access to health services. Thus, the clinical outcome in UCD vary in severity and over time from death shortly after birth, to different degrees of physical or cognitive impairment, to no symptoms until later in life.

Lastly, varying instruments used to assess biological, motor, or mental function across age groups can add, for each endpoint, between-instrument variability or instrument-specific measurement errors. For example, the UCD longitudinal study measures intelligence with several instruments, including the Wechsler preschool and primary scale intelligence [17] and the Wechsler Intelligence Scale for Children [18]. While age-sex standardization of each score ensures internal consistency, it does not guarantee comparability of scores between these two instruments in a study with preschoolers and adolescents.

### The benefits of designs with repeated measures

The role of rare disorder natural history studies or registries in informing clinical trials is well established [7]. Understanding the symptomatology and management of a rare disease over time and its natural history, informs multiple critical study design attributes. These attributes include the population inclusion and exclusion criteria, the study endpoints, and the times of initiation of a new therapy. They also inform the pre-specification of meaningful treatment effect size, frequency and timing of outcome assessments, and potential duration of follow-up. For example, the US CF Foundation registry has over 30,000 subjects, with extended follow-up for up to 20 years since 1986 [19]. The UCD consortium has also collected rich longitudinal data since 1996 with historical and prospective data on over 800 subjects covering the period from birth to adulthood [20]. As annual reports from these registries indicate, these epidemiologic natural history studies have informed the design of multiple prospectively planned studies investigating new therapies in CF and UCD.

Beyond understanding the natural history, longitudinal data collection or repeated assessments on the same individual enhance the ability to evaluate a disorder's impact over time. In a longitudinal, repeated measures design, the unit of analysis, whether randomized or observational, is a time period or a time point within a subject. In contrast, in a typical parallel-arm clinical study, a subject is the typical unit of analysis. Thus, when the population size is limited, the accruing of units of analyses with repeated measures can substantively enhance statistical power relative to between-subject comparisons, as discussed and illustrated by many authors [21–26].

Outcome measures on the same subjects are typically less variable than across subjects when the repeated measures are suitably spaced relative to disease progression and time of exposure to novel therapies (see “Considerations, advantages and limitations of longitudinal designs” section). Thus, in many situations longitudinal studies incorporating within-subject comparisons can estimate treatment effects more precisely than between-subject comparison in a parallel-arm or comparative cohort design.

Lastly, longitudinal observational designs are less prone to unmeasured confounding bias because they can control for non-time varying confounding, whether measured or unmeasured. Such non-time varying confounding includes important risk factors such as genetic mutation and medical history before diagnosis. In contrast, adjusting for confounding in cohort studies is only possible for measured characteristics.

## Randomized and observational study designs with repeated measure

This section reviews first those longitudinal designs relying solely on self-control to assess treatment effect, followed by designs augmenting external comparison with self-controlled comparisons. All these designs and analyses strategies are illustrated by examples in CF, UCD, or other rare disorders. For novel randomized or observational study designs, hypothetical examples are used.

### Self-controlled designs: relying on within-subject comparisons to estimate the causal effect

**Fig. 1:**
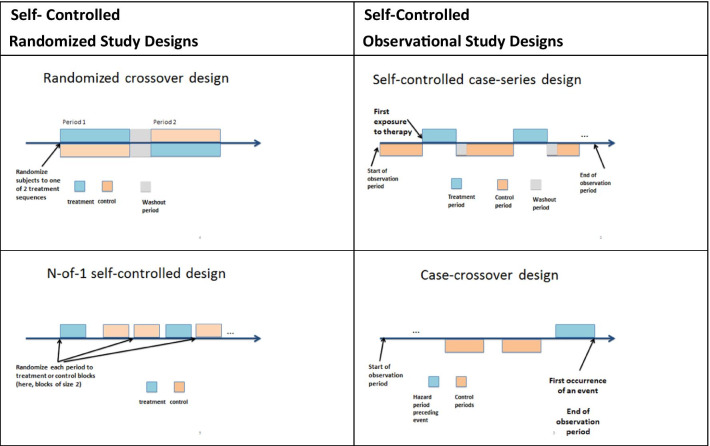
Self-controlled study design

A randomized, or observational, self-controlled study (Fig. 1) exploits time and controls for between-subject heterogeneity. These designs are feasible and meaningful when subjects can receive therapies in multiple distinct periods, and outcomes are responsive to change within a short time relative to treatment initiation.

Randomized self-controlled studies are well-known in rare disorders [22, 27]. In **the randomized crossover trial**, subjects contribute at least two time periods for outcome assessment and receive the novel therapy in one of these periods, in random order. For example, a pilot crossover study in CF compared pulmonary function improvements after treatment with different inhaled therapies, each for 3 weeks [28]. In the **randomized N-to-1 time-series designs**, one subject contributes multiple periods for outcome assessment, with a randomly assigned therapy in each period. For example, this design compared the efficacy of L-arginine capsules ingested weekly on reducing glutamine levels, a favorable outcome in UCD [29]. The observation periods in follow-up or look-back are of equal duration, typically. For example, in the CF case-crossover study, 3 weeks of treatment were separated by a 3-week washout period. In the UCDC 1-N study, periods were of the same duration of 1 week.

Observational studies using within-subject comparison are less well-known and potentially useful in rare disorders. The **self-controlled case-series design** is the non-randomized study parallel to the case-crossover or N-of-1 designs [30]. A self-controlled case-series is a relevant design for consideration to rare disease investigators because, compared to cohort studies, it has shown a remarkable ability to control for confounding in pharmacoepidemiology and comparative safety [31, 32]. This design only uses subjects who received both treatment and comparators at different periods and anchors the observation period to a subject’s initial treatment period. Thus, hypothetically, one could investigate l-arginine capsules' impact on reducing glutamine levels in UCD with an observational study of a sample of subjects with intermittent treatment with L-arginine and glutamine measurements in on and off exposure periods.

The **case-crossover design** is another potentially useful observational study design for consideration in rare disorders. It is nested in a case-series design and is particularly useful and cost-effective in investigating causes for rare dichotomous outcomes [33, 34]. This design investigates “the timing” of events rather than “the characteristics” of subjects with an event investigated in parallel-group designs. In this design, all subjects in the observational sample experienced the outcome, and exposure is ascertained in a hazard period immediately preceding the outcome and in control periods either preceding or following the hazard period. For example, hypothetically, if one wanted to investigate the benefit of a 4-week therapy in CF for prophylaxis of pulmonary exacerbations, one could recruit CF subjects after they experienced a pulmonary exacerbation and retrospectively collect exposure to therapies in a hazard period of 4 weeks preceding exacerbation compared to control periods, each of 4 weeks, before that.

### Sequential designs: augmenting between-group comparison with within-group comparisons

Sequential designs adapt their operating characteristics, such as therapy initiation or discontinuation time, investigated therapies, or study stopping time. Adaptation happens at sequential looks, based on information available at each look. Among these designs, we discuss those that augment between-subject comparison with within-subject comparisons. Although these designs' flexibility and efficiency are promising, most are novel in rare diseases or not as well established. Thus, few case studies exist of their successful application.

**Fig. 2:**
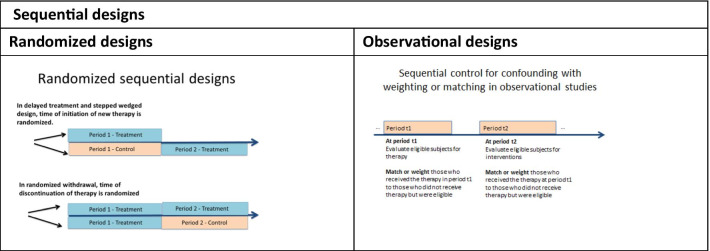
Sequential designs

**Sequential treatment initiation designs** (Fig. 2) include delayed treatment and stepped-wedged. In these designs, all subjects receive a new treatment, and the time of initiation is randomized. The **delayed treatment design** randomizes each subject to a specific initiation time. For example, the Phase III study of vestronidase alfa in patients with mucopolysaccharidosis VII [35] randomized participants to initiate treatments at week 1, week 9, week 17, or week 25 and followed subjects for 48 weeks. In a **randomized sequential withdrawal** (Fig. 2) study, all subjects initiate therapy, then discontinue therapy at a randomized time. For example, the Phase III study investigating the efficacy of pegvaliase in treating Phenylketonuria [36] randomized responders to therapy, determined after an assessment period, to either discontinue or continue therapy for 8 weeks.

The **stepped wedged design** (Fig. 2) randomizes each cluster of subjects to consecutive initiation times, where the cluster groups similar participants (e.g., geographic-based similarity) [37]. Clusters in stepped-wedged designs typically simplify the logistics of randomization. For example, a cluster-randomized study investigated the impact of a clinic-based behavioral intervention on adherence to medication in CF patients using each CF center as a cluster for randomization purposes [38].

The **sequential multiple assignment randomized trial** (SMART) adapts therapies or doses, as information about each subject’s response to therapy accumulates, at pre-specified interim analyses. Examples of their use exist in oncology and behavioral research [39, 40]. For example, the studies can start with multiple doses and change the investigated therapies at interim looks as information on subject’s response to treatment accumulates. Changes include dropping ineffective doses, adding a second-line therapy, or switching therapy of non-responders. Pre-planned sequential designs have the main advantage of allowing the study to stop early for efficacy or futility while calibrating these decisions for uncertainty at each look. One disadvantage of this approach is the increase in complexity of study planning and conduct.

The **longitudinal cohort study** can also leverage repeated measures on the same subject when the follow-up of each subject includes time on multiple treatments of interest, validated measures for the outcomes of interest, and time of initiation or duration of exposure or follow-up vary across subjects. When using this design for comparative safety or effectiveness, it can be re-imagined as the observational counterpart of a randomized study where the treatment decision is sequential, albeit not randomized [41].

For example, Li et al. [42] re-purposed the interstitial cystitis database as a design with sequential time from diagnosis to surgery initiation, cystoscopy, and hydrodistension. A recent comparative effectiveness study used this approach within the UCD Consortium longitudinal study to investigate liver transplantation's effectiveness on mortality and quality of life [43]. This approach divides the follow-up for each subject into multiple periods. Then, sequentially, in each period, subjects receiving the treatment are matched, on their medical history up to the start of each period, to eligible subjects who did not receive the treatment. Thus, a subject's off-therapy period can serve as the control for on-therapy periods.

Similarly, Hernan et al. re-imagined data from the Nurses’ Health Study [44] as a sequence of nested trials for hormone replacement therapy where the therapy initiation was sequential.

In this approach, subjects are dynamically weighted over time using the propensity score, defined as the probability of receiving treatment or control at a given time conditional on medical history at that time. Thus, the more similar the controls are to the treated subjects at a given time, the higher their weights. The treatment effect estimation incorporates these weights in the analysis.

## Considerations for valid causal inference with longitudinal data

### Causal inference, framework, and assumptions

Causal inference provides the framework for quantifying a new therapy's effect by comparing the observed outcomes under treatment received to the *potential outcomes* had the same subjects received an alternate therapy. The gold standard design for estimating a new therapy's treatment effect, including in rare diseases, is the randomized clinical trial. Because randomization ensures that patient groups are comparable or exchangeable before randomization, any differences in outcomes observed at the end of the trial can be causally attributed to treatment.

In observational studies, causal inference methods aim to minimize bias and confounding to ensure that differences in outcomes result from differences in treatment [45, 46]. These methods produce reliable treatment effect estimates under the assumptions of positivity (likelihood or propensity of receiving any treatment, is neither zero, nor one), exchangeability of treatments (comparability of characteristics between treatment groups), and consistency (equality of observed and potential outcomes). Practically, these assumptions apply to the units of analyses and ensuring that they are met or are plausible is design-specific as we describe in the next section.

### Design and analyses considerations with longitudinal studies for valid causal inference

#### Considerations, advantages and limitations of longitudinal designs

All studies considered in this paper have several advantages relative to the typical parallel-arm, placebo-controlled, randomized or cohort study. The main advantages of longitudinal studies, discussed in “The benefits of designs with repeated measures” section, are increasing the units of analyses, potentially reducing variability and confounding, and thus increasing study power to detect change. The longitudinal information can be in the follow-up or in the rich medical history. An additional advantage for randomized studies is that having all eligible subjects receive the new therapy reduces the ethical or recruitment concerns with having a placebo arm. While randomized studies can have strict inclusion and exclusion criteria, observational studies typically include a broader, more representative population with the potential for more generalizable findings. However, potential gains in study power from repeated measures are only possible when the causal inference assumptions above are met.

Of the three assumptions for valid causal inference, exchangeability of periods has broad implications on the feasibility and the specifications of longitudinal studies. In purely self-controlled studies, exchangeability is within-subjects, whereas, in longitudinal studies that combine person-time information across subjects and periods, exchangeability is relevant within and between subjects.

Randomization of subjects to different groups or therapies to different periods within the same subject guarantee positivity, some exchangeability, and lack of association of an outcome with future therapies. These three criteria are typically assumed in non-randomized studies comparisons and are more plausible in studies incorporating self-control than typical cohort studies. First, having a subset of subjects exposed to the treatment of interest and comparators guarantees positivity in observational studies. Second, by design, studies automatically control for non-time varying confounding, whether measured or unmeasured, when each subject serves as their control. Thus, the remaining threats to within-subject exchangeability include the carry-over effect of treatment in one period to the outcome on another period, time-varying confounding, time-varying treatments, or time-varying severity.

In practice, choosing short periods in longitudinal studies relative to age of participants and their disease progression, make the study feasible and exchangeability more plausible. For example, to investigate the effect of a novel therapy administered in the first few weeks of life in preventing brain injury, a study incorporating within-subject comparison would be more feasible for a brain function endpoint measured in the first few weeks of life (e.g., lab measurement or imaging) rather than later in life (e.g., neuropsychological tests after 3 years of age). Adding a short gap between treatment and control periods can also lessen concerns of the carry-over effect. For simplicity, periods are most often of the same duration. They typically start on the same day of the week and end on the same day of the week, or in the same season, to reduce the day of the week bias and seasonality.

Another challenge in using longitudinal observations for causal inference is determining a relevant *index date* for each subject that anchors pre-intervention medical history and post-intervention follow-up. An emerging approach in rare diseases is to use birth date or time of diagnosis as the index date. For example, in the earlier example investigating liver transplant effectiveness in patients with neonatal diagnosis in the UCD Consortium database [47], the index date was the birth date.

When selecting an observational database for longitudinal cohort studies, accuracy in timing is essential for multiple factors such as age, time of onset of symptoms, time of diagnosis, and developmental or therapeutic intervention milestones. The frequency of repeated measures is ideally compatible with exposure patterns, outcome natural history, and clinical visits pattern to increase adherence and minimize missing values in prospective studies.

#### Analysis considerations

Estimating a treatment effect in any of the above designs can vary in complexity based on the causal question and the study design. Reviews of analytical considerations in the rare diseases setting abound for randomized studies, for example in publications by these authors [48, 49]. Similar analytical considerations apply for observational studies with the added complexity of controlling for confounding when necessary. We summarize these considerations in this section based on the following characteristics: whether the study solely uses self-control or also uses between-subject comparison to estimate treatment effect, what confounding is adjusted for by design, and whether any time-varying or sequential adjustments are needed.

In self-controlled designs such as the crossover, N-of-1, or case series designs, estimating the treatment effect involves comparing outcomes during the treatment periods to outcomes in control periods. In a case-crossover design, one estimates the treatment effect by comparing treatments received during the hazard period immediately preceding the outcome to treatments received during control periods [33]. Under exchangeability, analyses estimating the treatment effect in purely self-controlled designs are paired analyses. They include paired t-tests or an F-test for continuous outcomes and a McNemar’s test for dichotomous outcomes. More complex analyses, such as hierarchical mixed effect models or conditional regression models, can adjust for order effect, time-varying confounding such as age, or outcome change over time, under additional assumptions [30, 50].

Sequential designs rely on longitudinal data collection to augment between-subject comparison with within-subject comparison from those that used more than one therapy. Randomized studies typically use hierarchical modeling or mixed effect modeling with a random effect accounting for correlation between repeated measures on the same subject [39]. With parametric models, use of hierarchical Bayesian models can also incorporate expert opinion and beliefs in prior distributions. Those update the model-based likelihood as data accrue and result in a treatment effect poster distribution [51].

In longitudinal cohort studies, analysis methods vary by the approach used to control for confounding including stratification, matching, weighting, or regression. They account for the correlation of multiple measurements from the same subject, weighting, and matching by including a random effect for subjects and using sandwich estimators, or bootstrap to derive standard errors. Using G-estimation or marginal structural models can handle time-varying treatment and control for time-varying confounding in the inference [52, 53]. For example, these methods have been used in secondary analyses of previously collected randomized clinical trials to evaluate an intervention that was not randomized [54]. In the rare disease setting, marginal structural models were used to evaluate a new therapy's efficacy and safety in severe juvenile dermatomyositis [55].

## Discussion

This paper gives an overview of randomized and observational study designs that exploit repeated measures in rare diseases to answer causal inference questions evaluating new or existing therapies. This paper illustrated some considerations for using these designs with case studies in the rare disease areas. While the focus of this paper is on rare disorders, many considerations and challenges apply broadly to causal inference methods in small samples. With the advent of genetic testing and personalized medicine, understanding how we can best estimate causal effects in small population subsets will be broadly relevant.

The longitudinal study designs we reviewed include self-controlled design and sequential designs. Ideally, the follow-up for each subject in these longitudinal studies would be long enough for observing exposure to therapy, in a critical time for therapeutic intervention on the outcomes of interest, and short enough to lessen the concern of time-varying confounding.

These designs have different operating characteristics than simple parallel designs that can make their conduct more feasible or information collection more efficient. Several algorithms exist to identify which randomized study design in rare diseases is useful based on the outcome severity, the rapidity of response to therapy, and ethical considerations around using a placebo arm [27, 56]. Based on our review in this paper, we argue that when it comes to using self-controlled observational studies or augmenting between-subject comparison with within-subject comparison, the same feasibility and adequacy principles developed for randomized studies apply for observational study designs. Additional complexities arise in observational studies to control for confounding between subjects and over time in the design and analysis. Control for confounding is achieved using weighting or matching methods developed for causal inference. Comparing how these methods perform in small samples based on different operating characteristics and, more specifically, the amount of confounding relative to within and between-subject variability would be valuable for rare diseases.

Rare disease networks show a great promise in accelerating our understanding of natural history and supporting the development of therapies in rare diseases [57]. The CF Foundation Therapeutics Development Network in the United States is the poster child of how such networks, when successful, can improve the lives of the patients they serve [23]. Several disease specific networks exist across the globe and include in the United States the National Institute of Health Rare Diseases Clinical Research Network [58] and the National Organization of Rare Diseases [59]. Novel sources for rare diseases data also include large electronic healthcare data networks such as the Patient-Centered Outcome Research Network [60]. Design considerations are possible to address in networks where the exchange of ideas and the economy of scale can lead to consensus clinical guidelines and standardizing data collection and capture.

## Data Availability

Not applicable.
